# A case of mistaken identity: An unusual presentation of neoplastic meningitis and a reminder of the hallmark features

**DOI:** 10.1002/ccr3.3289

**Published:** 2020-09-15

**Authors:** Catriona Davidson, Katerina Achilleos, Francesca Crawley, William Petchey

**Affiliations:** ^1^ Addenbrooke's Hospital Cambridge UK; ^2^ West Suffolk Hospitals NHS Trust Bury Saint Edmunds UK

**Keywords:** neoplastic meningitis, vertigo

## Abstract

This case offers an opportunity for education on the manifestations of neoplastic meningitis, a revision of the hallmark investigative features, and a reminder of the utilization of lumbar puncture in investigating unexplained neurological symptoms. Additionally, it emphasises the need for clinicians to avoid "diagnostic anchoring" when faced with recurrent attenders.

## Graphical abstract

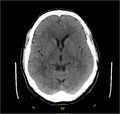

## 1 INTRODUCTION

Neoplastic meningitis remains a diagnostic challenge due to its varied clinical manifestations. We discuss a presentation with dizziness with an initial diagnosis of benign paroxysmal positional vertigo. We highlight the hallmark findings and reflect on how clinicians may miss evolving features in recurrent attenders due to "anchoring" to previous diagnoses.

Neoplastic meningitis, also known as leptomeningeal carcinomatosis or carcinomatous meningitis, occurs due to metastatic spread to the arachnoid and/or pia mater. It is associated with both hematological and solid malignancies, and its incidence is increasing.^1^ Whether this actually represents increasing recognition remains uncertain. The associated meningeal inflammation can lead to a wide variety of clinical presentations making diagnosis challenging. Previous case reports have detailed a range of clinical features including headache, altered mental status, and visual disturbances, often requiring repeated review prior to the eventual correct diagnosis of neoplastic meningitis being made.^2^, ^3^, ^4^ This case highlights another manifestation, namely dizziness, which was initially attributed to a diagnosis of benign paroxysmal positional vertigo (BPPV).

Although the prognosis of neoplastic meningitis is poor, early recognition and aggressive treatment can increase life expectancy.^5^ It is therefore important that clinicians are reminded of the varied presentations and the cardinal investigative findings that help make the diagnosis. Furthermore, this case hopes to serve as a reminder of the diagnostic challenge recurrent attenders present, and the risk of missing evolving symptoms due to fixation on initial diagnoses.

## 2 CASE PRESENTATION

### 2.1 History/Examination

**FIGURE 1:**
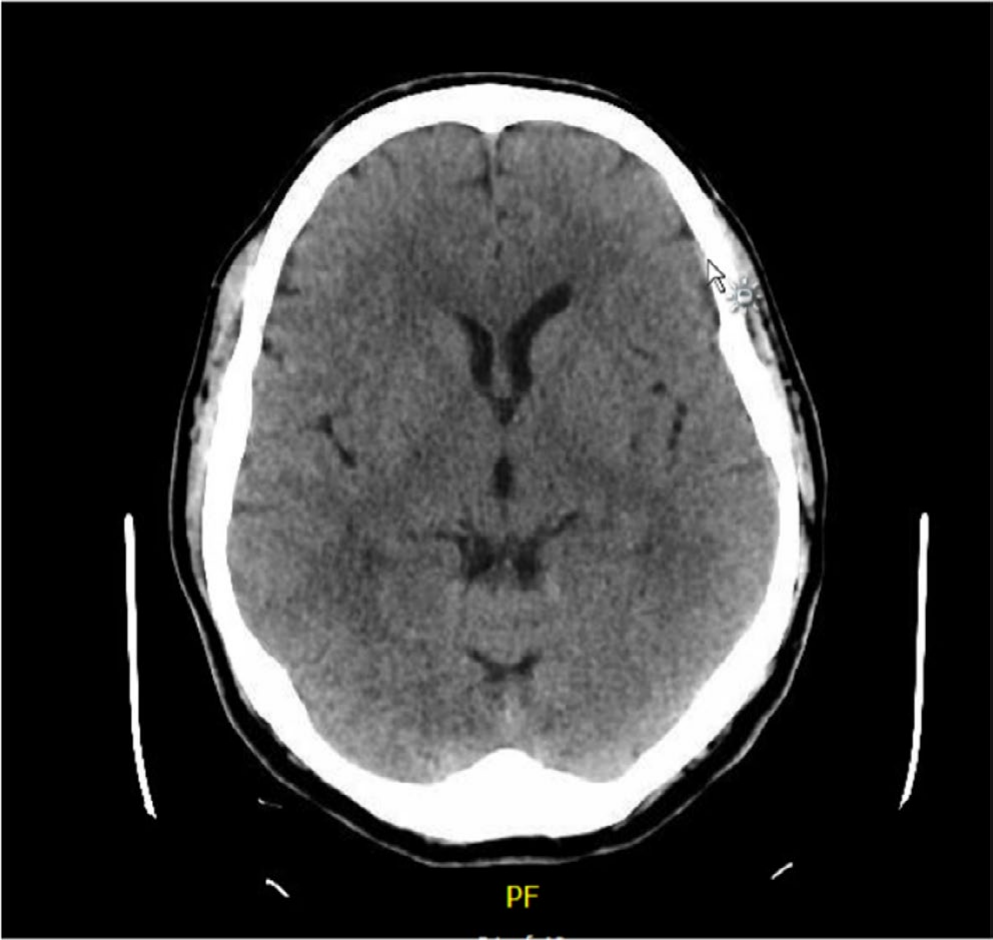
Transverse slice of initial CT Head on first presentation. Normal scan

**FIGURE 2:**
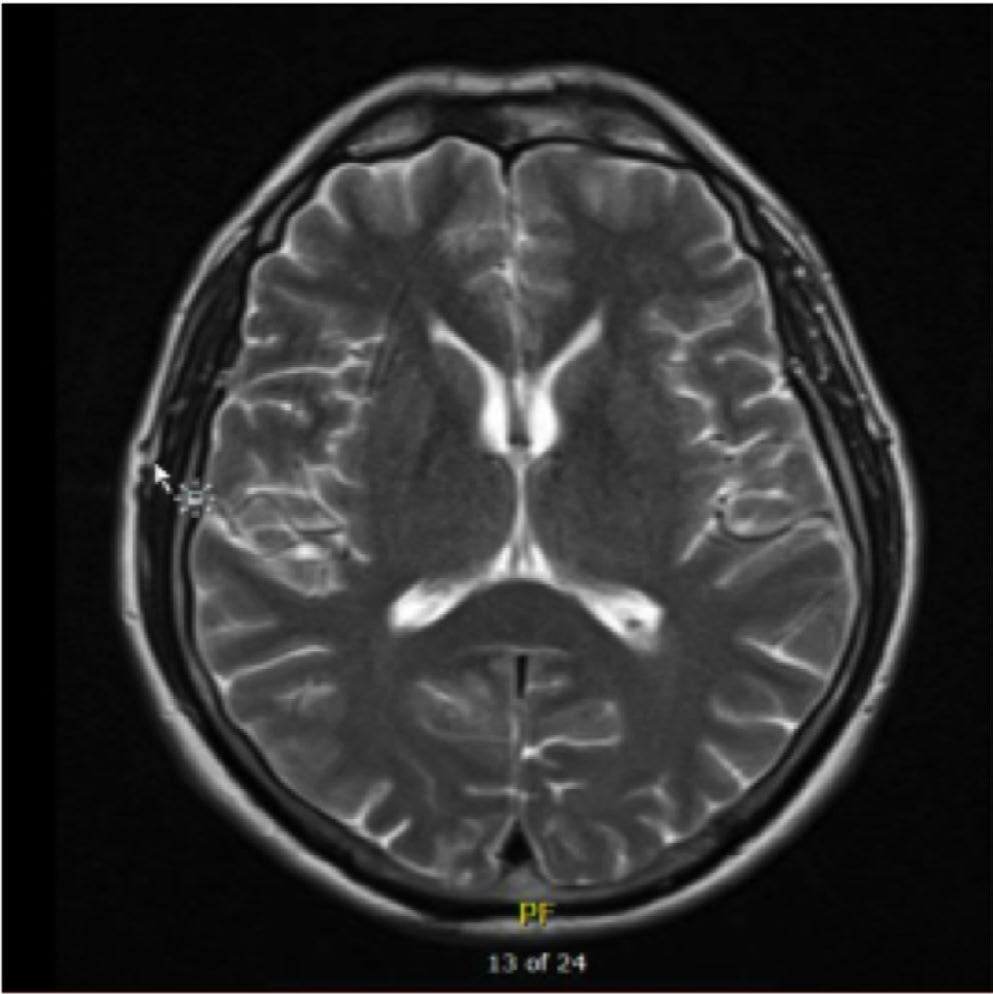
Transverse slice of initial MRI Head (T2 weighted) on first presentation. Normal scan

A 57‐year‐old male presented to the Emergency Department recurrently over a 4‐week period with dizziness, headache, and subsequent nausea and vomiting. He had no relevant past medical or travel history. He worked as a traffic warden, was a smoker with a 30‐pack year history, and consumed minimal alcohol. He had been investigated thoroughly on his first two presentations, having undergone a CT and MRI Head (Figures 1 and 2) and admission with assessment by a consultant neurologist. With a history descriptive of positional vertigo, torsional nystagmus on left lateral gaze and a positive Semont diagnostic maneuver (and resolution with Epley's maneuvers), he had been diagnosed with BPPV.

Following his diagnosis with BPPV, he presented two further times to the emergency department, being admitted on the fourth presentation due to significant dehydration and progressive weight loss, ascribed to the nausea and vomiting. His blood tests on this occasion demonstrated a significant neutrophilia, a raised urea (in keeping with clinical dehydration), and an unexplained elevated alkaline phosphatase (317 U/L). He described no additional symptoms, abdominal examination was unremarkable, and he did not display any new motor weakness. His diagnosis of BPPV was initially maintained until, on further exploration, he was found to now have features of cognitive impairment with an abbreviated mental test score of 6/10, and an inability to follow three stage commands. Further investigations were subsequently arranged.

### 2.2 Investigations

**FIGURE 3:**
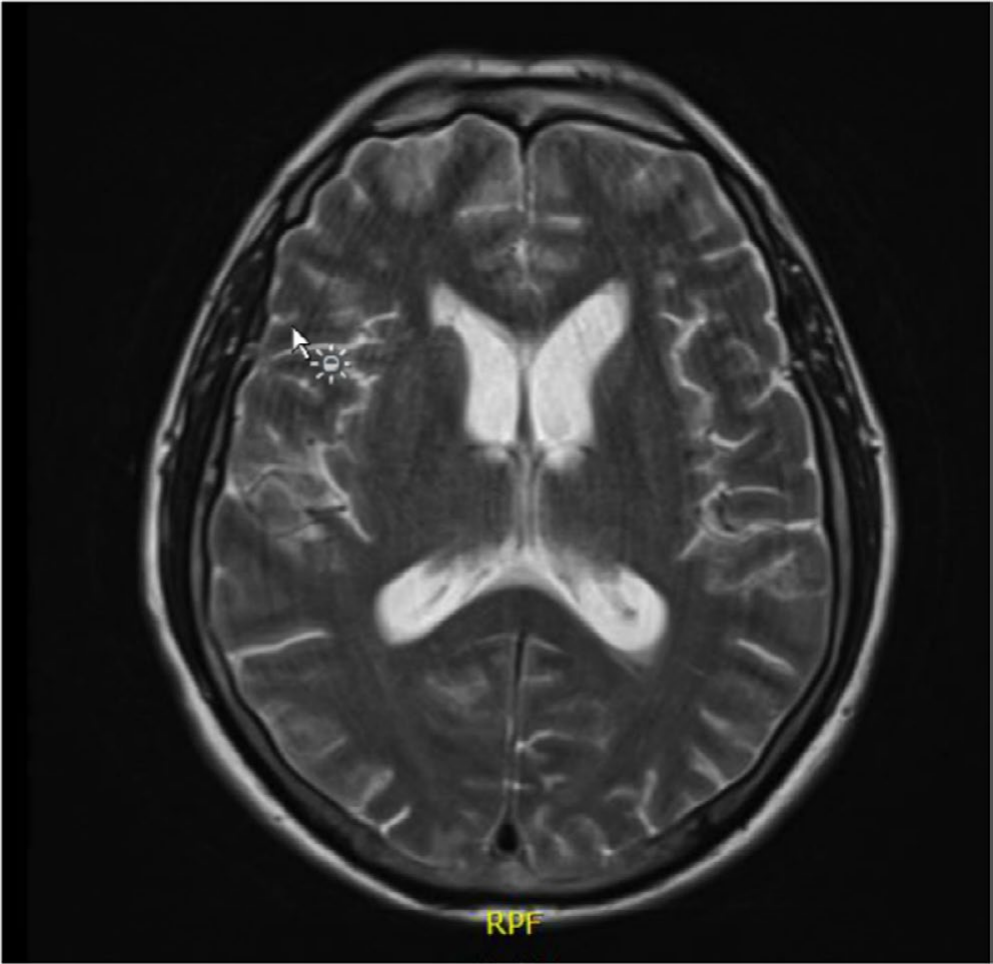
Transverse slice of repeat MRI Head (T2 weighted) on fourth presentation showing new hydrocephalus

**FIGURE 4:**
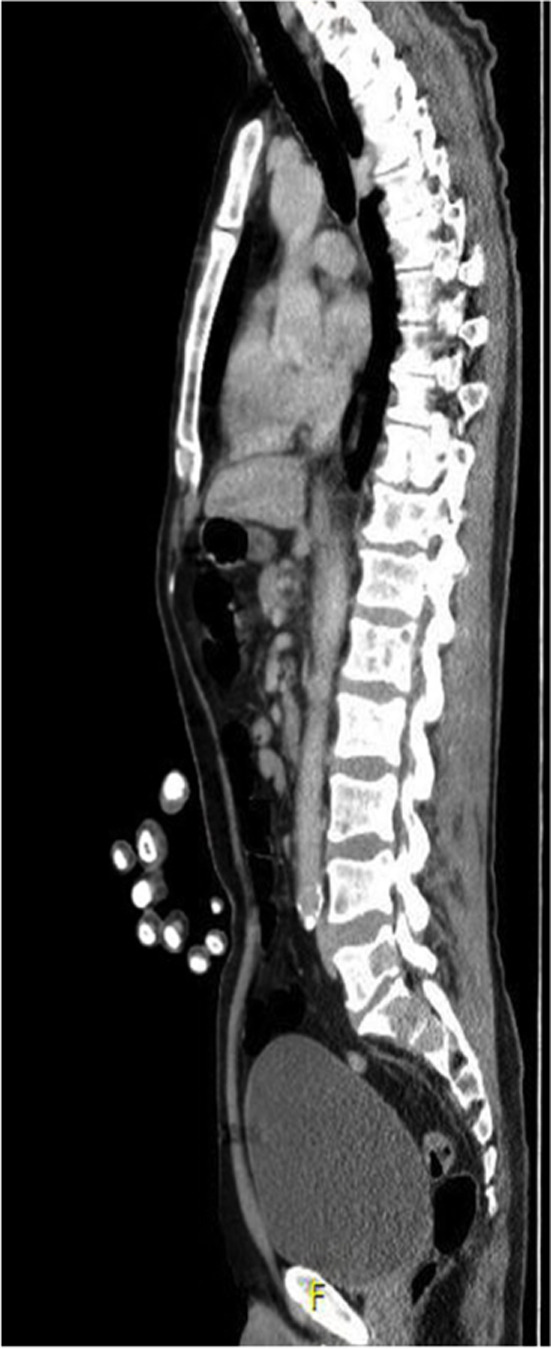
Sagittal section of CT Scan post‐CSF analysis, performed due to suspicion of neoplastic meningitis. Multiple vertebral metastases demonstrated

A repeat MRI Head was performed (Figure 3) that demonstrated mild hydrocephalus with no clear cause. A lumbar puncture (LP) was conducted, and analysis of the cerebral spinal fluid (CSF) showed an increased opening pressure, elevated white cell count (lymphocytic), high protein, extremely low glucose, and a high lactate (Table 1). Viral PCR, microscopy, bacterial, and fungal cultures all revealed no organisms. A CT scan of the chest, abdomen, and pelvis failed to identify a primary tumour; however, it revealed widespread metastatic bony deposits suggesting the diagnosis of neoplastic meningitis (Figure 4).

**Table 1:** Table depicting the CSF analysis of the patient in this case

CSF results (normal values^10^)	Values
Opening pressure (10‐20 mm H_2_O)	34 mm H_2_O
White cell count (0‐5 cells/µL)	43 × 10^6^
	100% lymphocytes
Protein (<0.45 g/L)	4.93 g/L
CSF Glucose (>60% serum glucose)	0.7
Serum Glucose	6.5
Lactate	9.9 mmol/L
Oligoclonal bands	Negative
MC&S, Viral PCR, 5D‐fungal cultures, AFB	Negative—no organisms seen
Xanthochromia	Not detected

### 2.3 Outcome/Follow‐up

After establishing the presumed diagnosis of neoplastic meningitis, a second high‐volume lumbar puncture was performed for CSF cytology, and a bone biopsy was arranged. Sadly, the patient suddenly deteriorated and required palliation. He died 4 days later.

Cerebral spinal fluid cytology, returned postmortem, demonstrated a poorly differentiated adenocarcinoma. Immunohistochemistry staining suggested this was of lung or upper gastrointestinal origin.

## 3 DISCUSSION

Neoplastic meningitis continues to be diagnostically challenging due to its subtle, varied, and nonspecific clinical features. This case illustrates an initial presentation of vertigo, with subacute progression to cerebral involvement and higher executive dysfunction. Headache remains the most common symptom of neoplastic meningitis (present in 66% of cases) but is nonspecific. 50% present with myelopathy, with symptoms such as lower limb weakness, paraesthesia, or bowel or bladder disturbance. 35% present with isolated or grouped cranial nerve palsies, and 15% present with cerebral dysfunction manifesting as higher cortical deficits such as confusion or dysphasia.^5^, ^6^ In retrospect, it is likely that the initial dizziness in this case was related to an isolated 8th (vestibulocochlear) nerve palsy with branch involvement leading to vestibular neuronitis.

Treatment of neoplastic meningitis is palliative and although cannot reverse existing neurological deficits may prevent deterioration.^7^ Aggressive treatment can increase survival time from 4 to 6 weeks to 6 months,^5^, ^6^, ^7^ highlighting the importance of early recognition and diagnosis. Clinicians must therefore be aware of the many manifestations and maintain a high index of suspicion.

With reference to diagnosis, a triad of factors including classical symptoms and signs, appropriate findings on MRI imaging, and CSF analysis should be used.^5^ MRI imaging is used to look for features of meningeal enhancement, whilst CSF findings of increased lymphocytes, high protein, very low CSF:serum glucose ratio, and high lactate, illustrated perfectly in this case, are highly suggestive of a diagnosis.^5^, ^6^ Positive cytology from CSF is gold standard although this cannot always be obtained and may require multiple, large volume LPs.^5^, ^6^ This case exemplifies the important role an LP can play in investigating new neurological symptoms.

Lastly, this case provides us with the opportunity to reflect on reasons behind diagnostic error. The prevalence of, and harm, that can arise from diagnostic error is well documented in the literature,^8^, ^9^ and it is therefore important that, as clinicians, we reflect on our practice in order to understand the factors influencing our decision making. This case offers a reminder of the particular challenge recurrent attenders present, and the bias that can arise from anchoring to an initial or previous diagnosis. This patient received a diagnosis of BPPV from a consultant neurologist and, despite changing clinical features on subsequent presentations, this diagnosis was maintained following review by three experienced general physicians. This highlights the importance of remaining open minded in such cases and recognizing diagnostic bias that may exist.

## CONFLICT OF INTEREST

None declared.

## AUTHOR CONTRIBUTIONS

CD and KA: performed as joint lead author. FC: performed as author. WP: performed as senior author.

## ETHICAL APPROVAL

Ethical approval was not required for this case report. Consent for publication was obtained.

## References

[1] Cancer Research UK . About carcinomatous meningitis 2014. https://www.cancerresearchuk.org/about‐cancer/secondary‐cancer/carcinomatous‐meningitis/about‐carcinomatous‐meningitis. Accessed August 20 2019.

[2] Foo CT , Burrell LM , Johnson DF . An unusual presentation of carcinomatous meningitis. Oxf Med Case Reports. 2016;2016(8):omw068. 27574561 10.1093/omcr/omw068 PMC5002064

[3] Trivedi T , Reddi R , Agarwal P , Arya A . A case of carcinomatous meningitis with occult primary malignancy. J Emerg Crit Care Med. 2018;2:21.

[4] Little J , Rajkumar C , Saleem W . A rare case of malignant meningitis from a likely bronchogenic primary cancer. Oxf Med Case Reports. 2019;1:omy114. 10.1093/omcr/omy114 PMC6345080 30697431

[5] Fields M . How to recognize and treat neoplastic meningitis. J Adv Pract Oncol. 2013;4(3):155‐160. 25031995 PMC4093424

[6] Chamberlain M . Neoplastic meningitis. Oncologist. 2008;13(9):967‐977. 18776058 10.1634/theoncologist.2008-0138

[7] Pellerino A , Bertero L , Ruda R , Soffietti R . Neoplastic meningitis in solid tumours: from diagnosis to personalised treatments. Ther Adv Neurol Diso. 2018;11:1756286418759618. 10.1177/1756286418759618 PMC5844521 29535794

[8] Scott I . Errors in clinical reasoning: causes and remedial strategies. BMJ Online June 2008. 2009;2:b1860. 10.1136/bmj.b1860 19505957

[9] Graber ML . The incidence of diagnostic error in medicine. BMJ Qual Saf. 2013;22:ii21‐ii27. 10.1136/bmjqs-2012-001615 PMC3786666 23771902

[10] Oxford medical education lumbar puncture CSF interpretation.

